# Effects of Melatonin Supplementation during Pregnancy on Reproductive Performance, Maternal–Placental–Fetal Redox Status, and Placental Mitochondrial Function in a Sow Model

**DOI:** 10.3390/antiox10121867

**Published:** 2021-11-24

**Authors:** Xie Peng, Xuelin Cai, Jian Li, Yingyan Huang, Hao Liu, Jiaqi He, Zhengfeng Fang, Bin Feng, Jiayong Tang, Yan Lin, Xuemei Jiang, Liang Hu, Shengyu Xu, Yong Zhuo, Lianqiang Che, De Wu

**Affiliations:** 1 Key Laboratory for Animal Disease Resistant Nutrition of the Ministry of Education, Animal Nutrition Institute, Sichuan Agricultural University, Chengdu 611130, China; pengxie@stu.sicau.edu.cn (X.P.); caixuelin@stu.sicau.edu.cn (X.C.); lijian@sicau.edu.cn (J.L.); huangyingyan@stu.sicau.edu.cn (Y.H.); liuhao@stu.sicau.edu.cn (H.L.); hejiaqi@stu.sicau.edu.cn (J.H.); fangzhengfeng@sicau.edu.cn (Z.F.); fengbin@sicau.edu.cn (B.F.); 13670@sicau.edu.cn (J.T.); linyan@sicau.edu.cn (Y.L.); jiangxuemei@sicau.edu.cn (X.J.); shengyuxu@sicau.edu.cn (S.X.); zhuoyong@sicau.edu.cn (Y.Z.); che.lianqiang@sicau.edu.cn (L.C.); 2 College of Food Science, Sichuan Agricultural University, Ya’an 625014, China; huliang@sicau.edu.cn

**Keywords:** melatonin, oxidative stress, placenta, mitochondria, fetal growth, sow

## Abstract

Melatonin (MT) is a bio-antioxidant that has been widely used to prevent pregnancy complications, such as pre-eclampsia and IUGR during gestation. This experiment evaluated the impacts of dietary MT supplementation during pregnancy on reproductive performance, maternal–placental–fetal redox status, placental inflammatory response, and mitochondrial function, and sought a possible underlying mechanism in the placenta. Sixteen fifth parity sows were divided into two groups and fed each day of the gestation period either a control diet or a diet that was the same but for 36 mg of MT. The results showed that dietary supplementation with MT increased placental weight, while the percentage of piglets born with weight < 900 g decreased. Meanwhile, serum and placental MT levels, maternal–placental–fetal redox status, and placental inflammatory response were increased by MT. In addition, dietary MT markedly increased the mRNA levels of nutrient transporters and antioxidant-related genes involved in the Nrf2/ARE pathway in the placenta. Furthermore, dietary MT significantly increased ATP and NAD^+^ levels, relative mtDNA content, and the protein expression of Sirt1 in the placenta. These results suggested that MT supplementation during gestation could improve maternal–placental–fetal redox status and reproductive performance by ameliorating placental antioxidant status, inflammatory response, and mitochondrial dysfunction.

## 1. Introduction

Rapid fetal growth during pregnancy leads to increased metabolic burdens on pregnant women or dams, causing elevated systemic oxidative stress [1,2]. Accumulating evidence suggests that maternal oxidative stress is associated with the occurrence of adverse pregnancy outcomes, such as preterm birth, preeclampsia, low birth weight, and fetal death [3,4]. The placenta is the only site for the transfer of nutrients to the fetus during gestation; thus, the placenta’s health and function are closely associated with the development of a healthy fetus [5]. However, the placenta is extremely sensitive to oxidative stress due to its high metabolic activity and extensive cell division [6]. In the placenta, DNA damage, lipid peroxidation, and protein denaturation caused by reactive oxygen species (ROS) can alter placental function, leading to a reduced capacity for the transfer of oxygen and nutrients to the fetus [7]. Dietary antioxidants can enhance the antioxidant status of gestating mammals, which has been considered to be an effective strategy to prevent adverse pregnancy outcomes [8].

Melatonin (MT) is primarily synthesized and released by the pineal gland, and has antioxidant, anti-apoptotic, and anti-inflammatory effects [9,10]. As a robust antioxidant, MT can directly scavenge ROS and also stimulate antioxidant enzymes. In addition to the pineal gland, the placenta has been considered as the major extrapineal organ of MT synthesis during gestation [11]. MT can easily and quickly pass across the placental barrier and enter fetal circulation, and is considered to be vital for placental functions and fetal growth [12]. Maternal MT supplementation during gestation has arisen as a plausible way to improve reproductive performance in several animal models. A previous study reported that MT could protect mice against lipopolysaccharide-induced intrauterine fetal death and IUGR via its antioxidant and anti-inflammatory properties [13]. Additionally, maternal dietary MT supplementation from mid-to late-gestation has been linked to alterations in utero-placental hemodynamics and amino acid flux, negating the consequences of IUGR in ewes [14,15]. Although MT has been shown to improve fetal growth by increasing uteroplacental blood flow and/or its antioxidant and anti-inflammatory effects, its underlying molecular mechanisms in placental growth and function have rarely been investigated.

With the progress of pregnancy, the placenta requires lots of energy to support rapid fetal growth, and mitochondria are critical as the primary sources of cellular energy [16]. Mitochondria are not only the main sites of ROS formation, but also a target of ROS attack, which may lead to changes in their function [17,18]. A previous report has found that maternal oxidative stress is closely related to placental mitochondrial dysfunction [19]. It has been suggested that placental mitochondrial dysfunction can affect subsequent fetal and placental growth [17,19]. Recently, Yang et al. reported that in aged oocytes, MT suppressed ROS production and reduced mitochondrial dysfunction [20]. However, there is limited research about the impacts of MT on placental mitochondrial function in pregnant mammals.

To our knowledge, no data are available currently regarding the effects of dietary MT supplementation during gestation on the reproductive performance and antioxidants status of sows, despite the fact that sows are increasingly used as animal models in biomedical researches on human pregnancy because of their similarity in terms of metabolic, inflammatory, gastrointestinal, and cardiovascular features [21]. Additionally, the placenta is a complex and transient organ that plays an important role in fetal development through its nutrients and hormone exchange functions between mother and fetus [22]. Placental dysfunction in human and sows has been implicated in disorders of maternal health and fetal growth [23,24]. Therefore, in this study, we hypothesized that dietary supplementation with MT in sow diets may improve reproductive performance by ameliorating maternal–placental–fetal redox status and placental mitochondrial dysfunction. The current study was carried out to verify the above hypotheses by evaluating the effects of MT on the reproductive performance, maternal–placental–fetal redox status, placental inflammatory response, and mitochondrial function.

## 2. Materials and Methods

The study was approved by the animal care and use committee of Sichuan Agricultural University (DKYB20131704).

### 2.1. Animals and Diet Design

A total of 16 Large White × Landrace fifth parity sows (3 years old) with similar backfat thickness were selected and inseminated with semen from the same Duroc boar. After artificial insemination, sows with their litters were randomly assigned to two treatment groups (*n* = 8 per group) and provided with a control diet (CON) or the same control diet containing 36 mg of MT (Sangon Biotech, Shangshai, China). Sows had similar starting weights between the two groups (CON: 225 ± 3.95 vs. MT: 216 ± 4.69 kg; *p* = 0.21). The dosage of MT was selected according to a previous study [25]. Feed was offered once daily at 14:00. Sows were fed 2.23 kg/d from mating until d 90 of gestation and 2.63 kg/d from d 91 of gestation until parturition. Sows were transferred to farrowing crates on d 107 of gestation.

MT was dissolved in absolute ethanol (12 mg/mL). The day prior to feeding, 3 mL of MT solution was absorbed onto 800 g of the CON diet in a plastic bag. The ethanol was allowed to evaporate overnight at room temperature and the individual plastic bags were sealed. The non-MT supplemented diet (800 g) was prepared in the same manner except that MT was not added to the ethanol. After the sows consumed the 800 g of modified feed, the remainder of the CON diet was given. In view of avoiding effects of lighting programs on the sows’ physiology and MT secretion (MT secretion is inhibited by light and stimulated by darkness), a lighting schedule of 12 h light and 12 h dark (darkness from 20:00 to 08:00) was used for the whole experiment. The control diet was formulated according to National Research Council (2012) recommendations [26]. The dietary ingredients and nutritional levels are listed in the Supplementary Materials, Table S1. All sows were allowed to drink water ad libitum throughout this study.

The numbers of total piglets born, alive, stillborn, and mummified, were recorded, and their individual weights were obtained at parturition. The number of low BW piglets (piglets born alive with weight < 900 g) was recorded. In addition, 16 new-born piglets (1 piglet per litter with the BW closest to the average BW of the litter) were selected before ingesting colostrum, and the placentas of selected piglets were collected.

### 2.2. Blood Sample Collection

Maternal blood samples (5 mL) were obtained from the ear vein on days 90 and 110 of gestation and on farrowing day. In addition, maternal ear vein blood samples (3 mL) were collected at 14:00 (prior to feeding), 17:00, and 20:00 (lights off) on day 102 of gestation. Each selected piglet was anaesthetized with sodium pentobarbital (30 mg/kg BW), and blood samples (5 mL) were obtained from the jugular vein. All blood samples were centrifuged at 3000× *g* at 4 °C for 15 min to obtain the serum, and immediately frozen at −80 °C for subsequent analyses.

### 2.3. Tissue Sample Collection

Sows were monitored continuously throughout parturition, and each piglet was matched to the corresponding placenta using the umbilical tagging procedure, as previously reported [27]. During sow farrowing, each umbilical cord was tied with a short silk line which was attached to a numbered tag to match the birth order of the piglets, so that when the umbilical retracted into the birth canal, it could easily be identified [19]. After placental expulsion and weight recording, approximately 3 g of placental tissue (4 to 5 cm from the cord insertion point) was collected, then immediately placed in liquid nitrogen and stored at −80 °C for subsequent analyses.

### 2.4. Analysis of Oxidative Stress Parameters in Serum and Placenta

The contents of total antioxidant capacity (T-AOC; catalogue no. A015-2-1) and malondialdehyde (MDA; catalogue no. A003-1-2), and the activities of glutathione peroxidase (GSH-Px; catalogue no. A005-1-2), catalase (CAT; catalogue no. A007-1-1) and superoxide dismutase (SOD; catalogue no. A001-1-2) in serum and placenta were determined using assay kits (Jiancheng Bioengineering Institute, Nanjing, China). Before the assays, the placental tissues were homogenized in ice-cold saline solution (1:9, *w*/*v*), and centrifuged at 3000× *g* for 10 min at 4 °C. The supernatants were collected for the analysis. The T-AOC was measured using the colorimetric method described by Wan et al. [28] and detected the absorbance value at 520 nm with colored and stable chelates when combined with phenanthroline. One unit (U) of T-AOC was defined as per milligram of tissue protein or per milliliter of serum with an increasing absorbance of 0.01 in 1 min. MDA concentrations were determined using the thiobarbituric acid method [29], which is based on the reaction of MDA with thiobarbituric acid to form a pink chromogen that can be spectrophotometrically determined at 532 nm. The GSH-Px activity was determined according to the method of Zhang et al. [30] by quantifying the rate of hydrogen peroxide-induced oxidation of reduced glutathione (GSH) to oxidized glutathione (GSSG). A yellow product, with absorbance at 412 nm, was formed on reaction of GSH with 5,5′-dithiobis-(2-nitrobenzoic acid). The CAT activity was measured by the method described by Ozmen et al. [31]. The enzymatic reaction was terminated by the addition of ammonium molybdate, which generated a light-yellow composite that could be measured at 405 nm. The SOD activity was measured spectrophotometrically at 550 nm according to the method of Jia et al. [32], and 1 U of SOD was defined as the quantity of enzyme required to produce 50% inhibition of nitric ion production. There was less than 5% variation of intra-assay and inter-assay coefficients for each assay.

### 2.5. Hormonal and Biochemical Parameters Analysis

To validate the ELISA, all assays included positive quality controls (QCs) and assays were only accepted if R^2^ was above 0.98, curve fit percentage recovery was within the 80–120% range, and intra-plate and inter-plate CV% had a threshold for acceptance below 20%. The serum concentrations of estradiol (E2; catalogue no. MM-048001) and progesterone (Prog; catalogue no. MM-120502) were determined using ELISA kits (Meimian Biotechnology, Nanjing, China). The minimal detection limit was 8 pmol/L for E2, 80 pmol/L for Prog. The intra- and inter-assay coefficients of variation were less than 10% and less than 12%, respectively. Concentrations of MT (catalogue no. RE54021) in the serum and placenta were measured with an ELISA kit (IBL, Hamburg, Germany). The sensitivity of this assay was 1.6 pg/mL. Both intra- and inter-assay coefficients of variation were less than 15%. Intra-and inter-assay CVs were less than 15. The serum concentrations of alanine aminotransferase (ALT; catalogue no. CH0101202), gamma-glutamyl transpeptidase (γ-GGT; catalogue no. CH0101204), and aspartate aminotransferase (AST; catalogue no. CH0101201) were measured with an automatic biochemical analyzer (Hitachi, Tokyo, Japan) according to corresponding commercial kits (Sichuan Maker Biotechnology Inc., Chengdu, China). ALT, AST, and γ-GGT were measured using an enzymatic rate method by ultraviolet and visible spectrophotometry. The minimal detection limit was 4 U/L for ALT, 3 U/L for AST, and 2 U/L for γ-GGT. There was less than 5% variation of intra-assay and inter-assay coefficients for each assay.

### 2.6. Measurement of Placental DNA, RNA and Protein

DNA, RNA, and protein were collected from snap-frozen placental samples (~0.1 g), using TRI Reagent RNA/DNA/Protein Isolation Reagent (Invitrogen Life Technologies, Carlsbad, CA, USA) and their concentrations were determined colorimetrically. DNA was analyzed fluorimetrically using the method of Prasad et al. [33]. RNA was determined by spectrophotometry using a modified Schmidt–Tannhauser method, as described by Munro and Fleck [34]. Protein concentration was analyzed according to the method of Lowry et al. [35] using reagents from Bio-Rad Laboratories (Hercules, CA, USA) and bovine serum albumin as the standard.

### 2.7. Measurement of ATP, NAD+, and NADH Levels in Placenta

Placental NAD^+^ and NADH levels were measured by the NAD^+^/NADH assay kit (catalogue no. S0175; Beyotime Biotechnology, Shanghai, China) according to the manufacturer’s instructions. The absorbance was determined using a microplate spectrophotometer (Spectramax 190, Molecular Devices, Sunnyvale, CA, USA) at a wavelength of 450 nm. The NAD^+^ and NADH levels were calculated according to the standard curve, and then, the ratio of NAD^+^/NADH was calculated. Placental ATP levels were measured using the enhanced ATP assay kit (catalogue no. S0026; Beyotime Biotechnology, China) according to the manufacturer’s instructions. ATP levels were calculated from relative light unit (RLU) values, which were measured using a GloMax 96 microplate luminometer (Promega, Stockholm, Sweden). Results were normalized to total protein concentration for inter-sample comparison.

### 2.8. Measurement of Mitochondrial Respiratory Chain Complex Activities

The activities of mitochondrial respiratory chain complexes I (catalogue no. FHTA-2-Y), II (catalogue no. FHTB-2-Y), and III (catalogue no. FHTC-2-Y) were measured with commercial kits (Suzhou Comin Biotechnology Co., Ltd., Suzhou, China). The activity of complex I was determined using the changing of NADH oxidation absorption at 340 nm. The activity of complex II was determined by calculating the alteration of the absorbance of 2,6-dichlorophenolindophenol at 605 nm. The activity of complex III was measured by calculating the alteration of the absorbance of cytochrome c at 550 nm.

### 2.9. Determination of Mitochondrial DNA (mtDNA) Content

The content of mtDNA relative to nuclear genomic DNA was determined by co-amplification of the mt d-loop and the nuclear-encoded β-actin using real-time PCR according to our previous study [36]. Total DNA of frozen placental tissue was extracted with DNAiso reagent (catalogue no. DP304; Tiangen Biotech, Beijing, China). The DNA samples were adjusted to a concentration of 100 ng/μL. The amounts of mt d-loop and β-actin gene were quantified by fluorescent probes. The primers and probe sequences are listed in the Supplementary Materials, Table S2. PCR amplification was carried out in a 20 μL total volume consisting of 1 μL DNA template (100 ng), 1 μL enhance solution, 1 μL probes, 8 μL TaqMan Universal Master Mix, 1 μL forward primer, 1 μL reverse primers, and 7 μL double-distilled H_2_O. The fluorescence spectra were monitored with a Real-Time PCR Detection System (ABI 7900HT, Applied Biosystems, Foster City, CA, USA) as follows: 95 °C for 10 s, 50 cycles involving a combination of 95 °C for 5 s and 60 °C for 25 s, and 95 °C for 10 s. The 2^−ΔΔCt^ method was used to calculate the relative mtDNA content [37].

### 2.10. Measurement of Inflammatory Cytokine in Placenta

Approximately 0.1 g of placenta tissue samples were homogenized in 0.9% ice-cold physiological saline (1:9, *w*/*v*), then centrifuged at 3500× *g* at 4 °C for 15 min. The supernatant was collected to determine the concentrations of tumor necrosis factor α (TNF-α; catalogue no. MM-038301), interleukin 6 (IL-6; catalogue no. MM-041801), and IL-8 (catalogue no. MM-041701) with commercial ELISA kits (Meimian Biotechnology, Nanjing, China). The minimal detection limit was 10 pg/mL for TNF-α, 50 ng/mL for IL-6, and 15 ng/mL for IL-8. The intra-and inter-assay coefficients of variation were less than 10% and less than 12%, respectively. Results were normalized to total protein concentration for inter-sample comparison.

### 2.11. RNA Extraction and Gene Expression Analysis

Total RNA of frozen placental tissue was extracted with Trizol reagent (catalogue no. 9109; TaKaRa, Dalian, China). RNA integrity was checked by electrophoresis on a 1.0% agarose gel, and RNA quality and concentration were measured using a NanoDrop ND-2000 spectrophotometer (Thermo Fisher Scientific, Wilmington, DE, USA). Total RNA (1 μg) was reverse transcribed into complementary DNA using the PrimeScript RT Reagent Kit (catalogue no. RR047A; TaKaRa, Dalian, China). Real-time quantitative PCR was performed using SYBR Green (catalogue no. RR820A; TaKaRa, Dalian, China) with ABI-7900HT (Applied Biosystems, Foster City, CA, USA). The primers are listed in the Supplementary Materials, Table S3. The reaction mixture of 10 μL included 5 μL of SYBR Premix Ex Taq (2×), 0.4 μL of forward primer (10 μmol/L), 0.4 μL of reverse primer (10 μmol/L), 0.2 μL of ROX reference dye (50×), 1 μL of cDNA, and 3 μL of double-distilled water. The PCR procedure was as follows: pre-denaturating at 95 °C for 30 s, 40 cycles of denaturation at 95 °C for 5 s, annealing at 60 °C for 34 s, and a final extension at 72 °C for 6 min. β-actin was used as an internal control, and the data were calculated using the 2^−ΔΔCt^ method.

### 2.12. Western Blot Analysis

Frozen placental tissue samples were homogenized in liquid nitrogen and lysed in cell lysis buffer (Beyotime Biotechnology, Shanghai, China). Protein content was measured with the BCA kit (Beyotime Biotechnology, Shanghai, China). The Western blot analysis steps were conducted according to previously reported methods [38]. Primary antibodies against SIRT1(1:1000, 9475S, CST, Danvers, MA, USA) and GAPDH (1:1000, abs132004, Absin, Shanghai, China) were used in this study. Blots were analyzed with ImageJ software (NIH, Bethesda, MD, USA).

### 2.13. Statistical Analysis

An individual sow or piglet was considered as the experimental unit. The statistical analysis was performed using the SAS statistical software 9.2 (SAS Institute, Cary, NC, USA). The rate of low birthweight piglets (BW < 900 g) was calculated with the chi-square test. The normal distribution of the other data in this study was calculated with the Shapiro–Wilk test, followed by Student’s *t*-test. Data were presented as means ± SEM. Significant differences were set at *p* ≤ 0.05, and a tendency was considered when 0.05 < *p* < 0.10.

## 3. Results

### 3.1. Reproductive Performance

**Figure 1:**
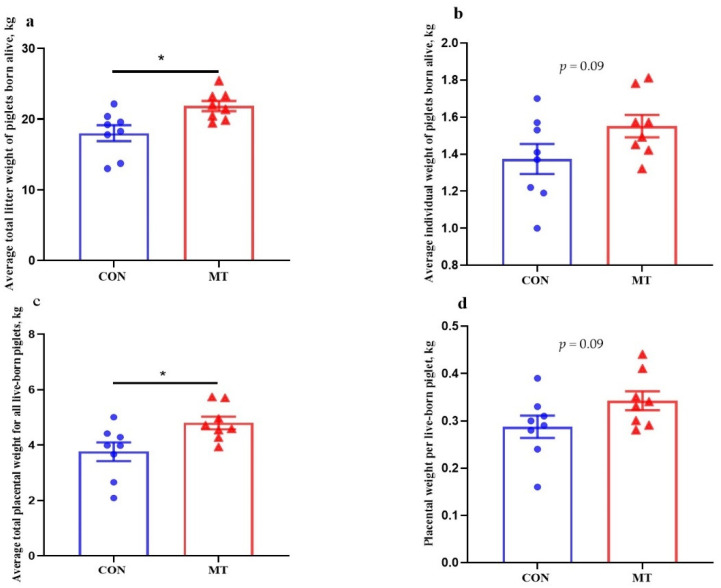
Effects of maternal MT supplementation during gestation on litter performance and placental weights. (**a**) Average total litter weight of piglets born alive. (**b**) Average individual weight of piglets born alive. (**c**) Average total placental weight for all live-born piglets. (**d**) Placental weight per live-born piglet. Data are presented as means ± SEM, *n* = 8. * *p* < 0.05. CON = control, MT = melatonin.

As shown in Table 1, no differences were found in the average litter size of the total of piglets born, live-born, stillborn, and mummified piglets between the two groups (*p* > 0.05). Maternal MT supplementation reduced (*p* < 0.05) the percentage of piglets born alive with weight < 900 g compared with the CON. The average total litter weight of live-born and the average total placental weight for all live-born piglets were increased (*p* < 0.05) in the MT group (Figure 1a,c). Meanwhile, the average individual piglet weight of live-born piglets (*p* = 0.09) and the placental weight per live-born piglet (*p* = 0.09) tended to increase in the MT group (Figure 1b,d).

### 3.2. Hormonal and Biochemical Parameters in Serum and Placenta

**Figure 2:**
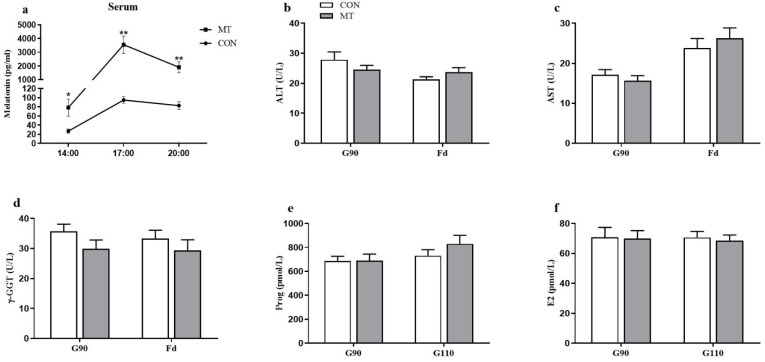
Effects of maternal MT supplementation during gestation on hormonal and biochemical parameters in the serum and placenta. (**a**) MT concentration in maternal serum on d 102 of gestation (*n* = 7/group). (**b**) ALT concentration, (**c**) AST concentration, (**d**) γ-GGT concentration, (**e**) Prog concentration, and (**f**) E2 concentration in maternal serum (*n* = 8/group). Data are presented as means ± SEM. * *p* < 0.05, ** *p* < 0.01. CON = control, MT = melatonin, G90 = d 90 of gestation, G110 = d 110 of gestation, Fd = farrowing day, ALT = alanine aminotransferase, AST = aspartate aminotransferase, γ-GGT = gamma-glutamyl transpeptidase, Prog = progesterone, E2 = estradiol-17β.

As shown in Figure 2a, maternal MT supplementation significantly elevated serum MT concentrations at all time points tested on d 102 of gestation (G102), and the highest serum MT concentration occurred at 17:00 relative to the two other timepoints examined (*p* < 0.01). However, the serum concentrations of ALT, AST, and γ-GGT on G90 and farrowing day (Fd) did not differ between the two groups (Figure 2b–d). Besides, the serum concentrations of Prog and E2 on G90 and G110 (*p* > 0.05) were not influenced by MT supplementation (Figure 2e,f).

### 3.3. Antioxidant Capacity in Serum of Sows and New-Born Piglets

**Figure 3:**
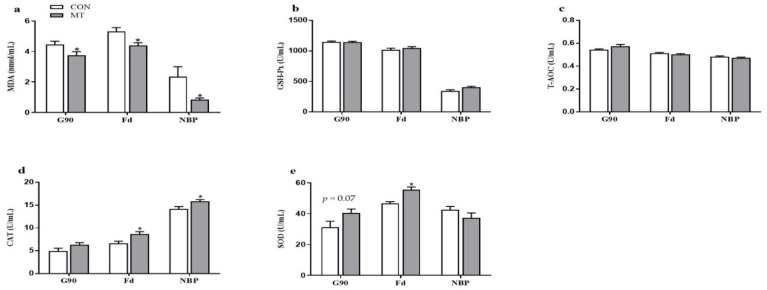
Effects of maternal MT supplementation during gestation on antioxidant status in serum of sows and new-born piglets. (**a**) MDA concentration, (**b**) GSH-Px activity, (**c**) T-AOC concentration, (**d**) CAT activity and (**e**) SOD activity in the serum of sows and new-born piglets. Data are presented as means ± SEM, *n* = 8. * *p* < 0.05. CON = control, MT = melatonin, G90 = d 90 of gestation, Fd = farrowing day, NBP = new-born piglets.

The content of MDA in the serum of sows at G90 and Fd and in NBP (new-born piglets) was decreased (*p* < 0.05) by MT supplementation (Figure 3a). The activity of GSH-Px and the content of T-AOC in serum of sows and NBP were not statistically different between the two groups (*p* > 0.05) (Figure 3b,c). CAT activity in the serum of sows at Fd and in NBP was increased (*p* < 0.05) in the MT group (Figure 3d). In addition, maternal MT supplementation increased (*p* < 0.05) SOD activity in the serum of sows at Fd, and tended to increase (*p* = 0.07) at G90 (Figure 3e).

### 3.4. Antioxidant Status in the Placenta

**Figure 4:**
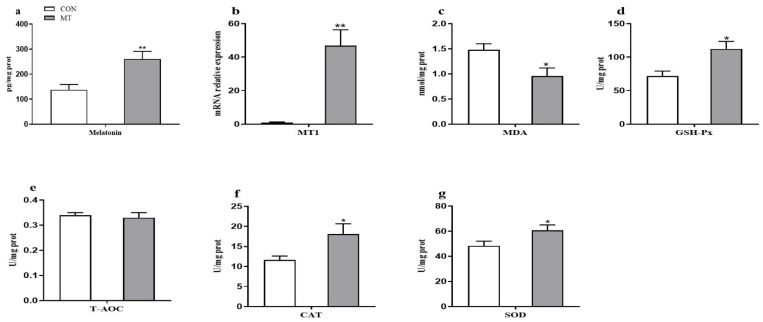
Effects of maternal MT supplementation during gestation on antioxidant status in the placenta. (**a**) MT concentration in placenta. (**b**) mRNA relative expression of MT1. (**c**) MDA concentration. (**d**) GSH-Px activity. (**e**) T-AOC concentration. (**f**) CAT activity. (**g**) SOD activity. Data are presented as means ± SEM, *n* = 8. * *p* < 0.05, ** *p* < 0.01. CON = control, MT = melatonin, MT1 = melatonin receptor 1A.

As shown in Figure 4a,b, the placental MT concentration and the mRNA expression level of MT1 were increased by MT supplementation (*p* < 0.01). However, the content of MDA in the placenta was reduced (*p* < 0.05) by MT supplementation (Figure 4c). The GSH-Px, CAT, and SOD activities in the placenta were increased (*p* < 0.05) in the MT group (Figure 4d–g), while the content of T-AOC did not differ between the two groups (Figure 4e).

### 3.5. Placental DNA, RNA, and Protein Concentrations

As shown in Table 2, dietary MT significantly increased (*p* < 0.01) the placental protein/DNA ratio and tended to increase (*p* = 0.07) placental RNA/DNA ratio and protein concentration. However, the DNA and RNA concentrations in the placenta did not differ between the two groups.

### 3.6. Placental ATP Levels and Mitochondrial Function

**Figure 5:**
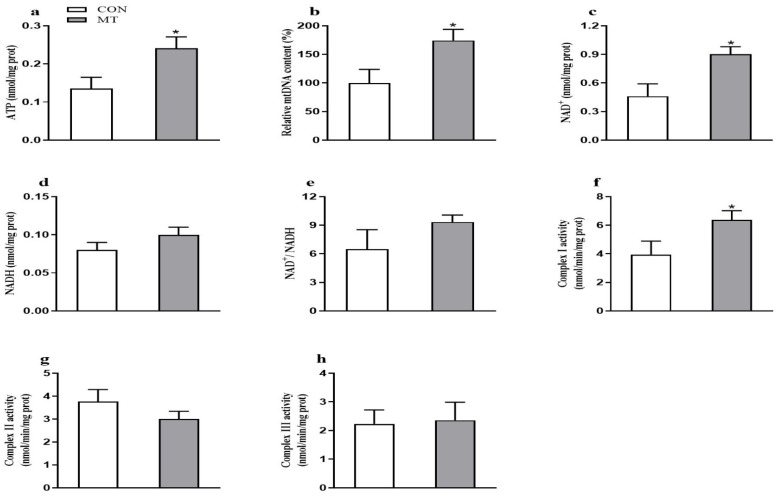
Effects of maternal MT supplementation during gestation on mitochondrial function in the placenta. (**a**) ATP levels. (**b**) Mitochondrial DNA (mtDNA) copy number. (**c**) NAD^+^. (**d**) NADH. (**e**) The ratio of NAD^+^/NADH. (**f**) Complex I activity. (**g**) Complex II activity. (**h**) Complex III activity. Data are presented as means ± SEM, *n* = 8. * *p* < 0.05. CON = control, MT = melatonin.

**Figure 6:**
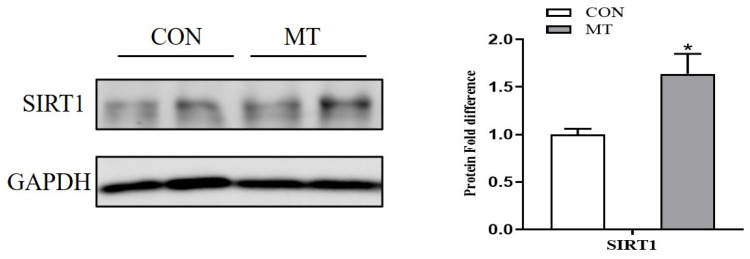
Effects of maternal MT supplementation during gestation on Sirt1 protein expression in placenta. Data are presented as means ± SEM, *n* = 6. * *p* < 0.05. CON = control, MT = melatonin.

As shown in Figure 5, the placental ATP and NAD^+^ levels (Figure 5a,c), the relative mtDNA content (Figure 5b), and complex I activity (Figure 5f) were increased (*p* < 0.05) by MT supplementation. Meanwhile, the protein expression level of SIRT1 in the placenta were also increased (*p* < 0.05) in the MT group (Figure 6). However, the NAD^+^/NADH ratio, NADH level, and complex I and III activities in the placenta did not differ between the two groups.

### 3.7. Placental Inflammatory Cytokine Concentrations

**Figure 7:**
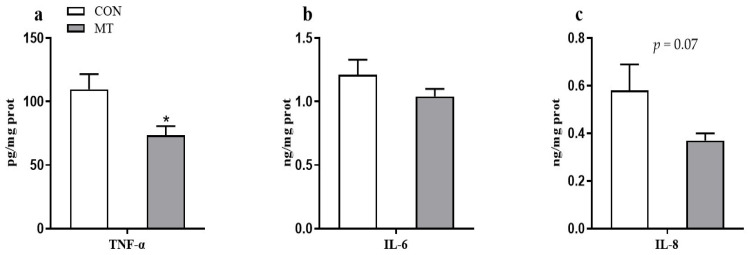
Effects of maternal MT supplementation during gestation on inflammatory cytokine in placenta. (**a**) TNF-α concentration. (**b**) IL-6 concentration. (**c**) IL-8 concentration. Data are presented as means ± SEM, *n* = 8. * *p* < 0.05. CON = control, MT = melatonin.

As shown in Figure 7, dietary MT significantly decreased (*p* < 0.01) the concentration of placental TNF-α (Figure 7a) and tended to decrease (*p* = 0.07) the concentration of placental IL-8 (Figure 7c). However, the concentration of IL-6 in the placenta did not differ between the two groups (Figure 7b).

### 3.8. Nrf2-Regulated Gene Expression in the Placenta

**Figure 8:**
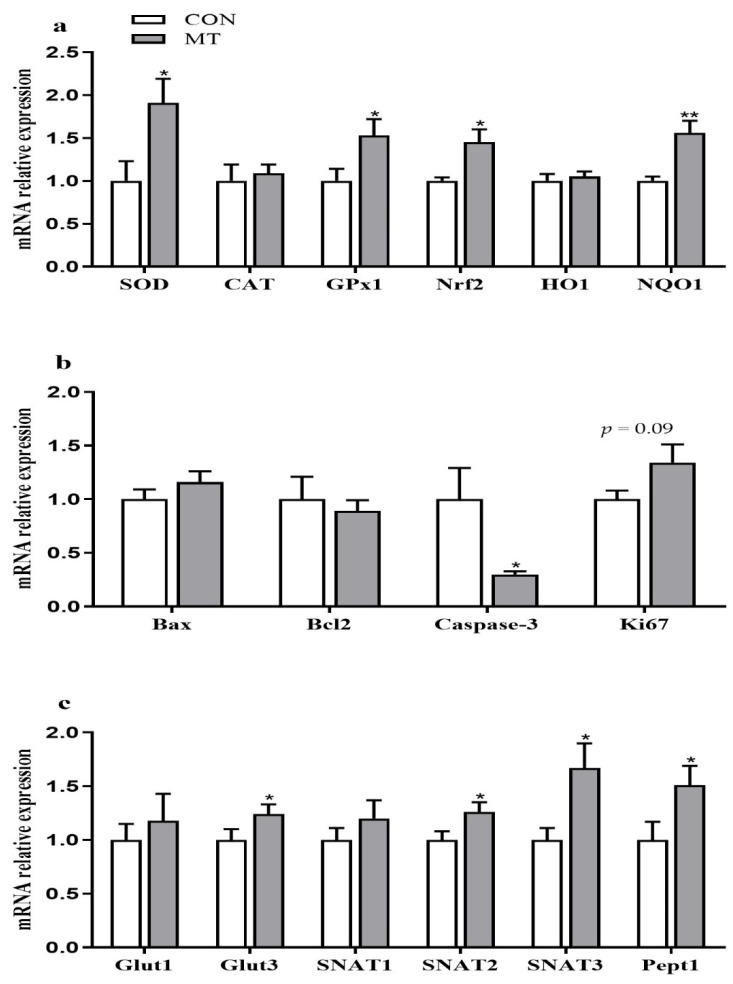
Effects of maternal MT supplementation during gestation on the relative expression levels of critical genes involved in the placental functions. (**a**) Nrf2-regulated gene expression. (**b**) Apoptosis and proliferation-related gene expression. (**c**) Nutrient transporter gene expression. Data are presented as means ± SEM, *n* = 8. * *p* < 0.05, ** *p* < 0.01. CON = control, MT = melatonin, Glut1 = Slc2a1, Glut3 = Slc2a3, SNAT1 = Slc38a1, SNAT2 = Slc38a2, SNAT3 = Slc38a4.

The mRNA expression levels of *Nrf2*-regulated genes are presented in Figure 8a. Maternal MT supplementation increased (*p* < 0.05) the mRNA levels of *SOD*, *GPx1*, *Nrf2*, and *NQO1* in the placenta.

### 3.9. Apoptosis and Proliferation-Related Gene Expression in the Placenta

Maternal MT supplementation decreased (*p* < 0.05) the mRNA level of *Caspase-3*, while it tended to increase (*p* = 0.09) that of *Ki67* in the placenta (Figure 8b). There were no differences in mRNA levels of *Bax* and *Bcl2* between the two groups (*p* > 0.05).

### 3.10. Nutrient Transporter Gene Expression in Placenta

The mRNA expression levels of nutrient transporter genes, including *Glut3*, *SNAT2*, *SNAT3*, and *Pept1*, were increased by MT supplementation (Figure 8c). However, the mRNA levels of *Glut1* and *SNAT1* did not differ between the two groups.

## 4. Discussion

MT is a safe molecule with low toxicity. It has been reported that MT is adequately safe to be administered during pregnancy, even in high doses (up to 200 mg/kg/day) [39]. In this study, dietary supplementation with MT significantly elevated serum melatonin concentrations at all time points tested, even prior to the onset of feeding. The patterns of circulating MT in pigs have been described as unchanging, irregular, or nocturnal [40,41,42]. The inconsistency in the MT profiles could be related to differences in assay methodology, geographical location of the study, lighting regimen, acclimation period, and method for administration of MT [42,43,44,45]. So far as we know, there are no published reports from sequential blood sampling in pregnant sows fed such a high dose of MT to compare with our study. In domestic gilts, oral application of 1 mg MT increased plasma MT concentrations within 30 min and that these remained high for at least 8 h [46]. In addition, oral application of MT (3 mg/d) at 15:00 h showed an elevated peak of melatonin ~6 h after lights off, and the overall patterns for MT in circulation appeared episodic at 3–6 h intervals while lights were on [42]. Furthermore, in this study, no statistically significant differences of MT supplementation on serum ALT, AST, and γ-GGT concentrations are an indication that the treatments have no obvious effect on liver function. Moreover, Prog and E2 are important regulators of reproduction, which play a crucial role in establishing and maintaining pregnancy [47,48]. In the current study, serum concentrations of Prog and E2 were not altered by MT supplementation. The findings of our study were not consistent with the previous results obtained in mice [49], which showed that intraperitoneal injection of MT (15 mg/kg) significantly decreased E2 concentration, with no obvious effects on Prog at day 6 of gestation. In addition, a previous study showed that MT dose- and time-dependently increased Prog production in the cultured luteal cells of pregnant sows [50]. Previous study also reported that suitable doses of MT (10^−8^, 10^−7^, and 10^−6^ M) could promote Prog secretion in cultured pig luteal cells, whereas a higher concentration of MT (10^−5^ M) exhibited no obvious difference between the groups [51]. Together, the discrepancies between studies suggest that the effect of melatonin on steroid hormone secretion could be highly complex, which might be explained by a number of factors, such as animal species, physiological conditions, as well as the dose and the duration of MT supplementation. However, the underlying mechanisms need further investigation. Based on the findings in the current study, it appears that the administration of 36 mg MT in pregnant sows showed no adverse maternal effects on the health status and secretion of reproductive hormones.

Melatonin works in a variety of ways as a circadian rhythm modulator, immunomodulator, direct free radical scavenger, and indirect antioxidant and cytoprotective agent in the maternal–placental–fetal unit, and it seems to be crucial for successful pregnancy [52,53]. Increasing evidence supports the idea that therapeutic use of melatonin during pregnancy may reduce materno-fetal complications and prevent neonatal diseases [12,54]. Oxidative stress, resulting from an antioxidant–prooxidant imbalance, has been implicated in the initiation or development of reproductive diseases (e.g., IUGR) affecting female reproductive processes [55]. Pregnancy is a state of high oxidative stress in humans and livestock, which is deleterious to placental development and fetal growth [56]. MDA is a primary marker of lipid peroxidation caused by ROS [57]. CAT, SOD, and GPX are important enzymes that constitute a first line antioxidant defense system to scavenge ROS [58]. In this study, maternal MT supplementation improved antioxidant status to a certain degree, and reduced MDA content in the serum of sows and new-born piglets. The new-born piglets were slaughtered before suckling colostrum in our study, which implied that the antioxidant defense capacity of new-born piglets may be enhanced in utero. During pregnancy, MT in maternal blood can easily pass across the placenta into fetal circulation and affect the fetus directly [52]. MT (10 mg/kg) administration to pregnant rats has been demonstrated to improve antioxidant activity and to protect against oxidative mitochondrial damage in the fetal rat brain [59,60]. In addition, pharmacological doses of melatonin (ranging from 0.1 to 4.0 mM) could reduce MDA content in rat brains in in vitro conditions [61]. Thus, the present results indicating that antioxidant activity in sows and new-born piglets was increased by dietary MT supplementation.

In general, maternal serum MT concentrations gradually increase during pregnancy, and this is mainly ascribed to placental production [11]. Moreover, high MT levels in the human placenta have been observed even during daytime in normal pregnancy, and lower placental MT levels were detected in pregnancies complicated by preeclampsia compared to normal pregnancies [52,62,63]. Our results showed that maternal MT supplementation increased placental MT concentrations and melatonin receptors, suggesting a beneficial effect of oral administration of MT in improving placental–fetal development. Supportively, MT has been reported to protect the villous trophoblast against hypoxia/reoxygenation-induced oxidative stress and proposed as a potential preventive option for IUGR [64]. In addition, our results showed that maternal MT supplementation tended to increase the average weight of live-born piglets, and markedly reduced the percentage of lower birth weight piglets (BW < 900 g). Similarly, a previous report demonstrated that MT supplementation during early-to mid-gestation can increase fetal weight at d 50 of gestation in gilts [25]. Additionally, consistent with the previous study that MT supplementation in undernourished pregnancy restored birth weight by increasing placental antioxidant enzymes [18], our data showed that GSH-Px, SOD, and CAT activities in the placenta were upregulated, and the content of MDA was decreased due to MT supplementation. Furthermore, a recent study showed that MT could attenuate intrauterine inflammation-induced placental oxidative stress via activating the Nrf2/ARE pathway [9]. Nrf2 performs a critical role in regulating antioxidant enzymes and phase II detoxification enzymes by the transcriptional activation of many genes containing ARE [65]. NQO1 is a detoxification enzyme that reduces NADPH oxidase activity and ROS production [66]. In this study, dietary supplementation with MT significantly increased the mRNA levels of antioxidant-related genes involved in the Nrf2/ARE pathway (*Nrf2*, *SOD*, *GPx1*, and *NQO1*). Therefore, these results may suggest that MT could promote fetal growth at least partly through its reduction of placental oxidative stress via activating the Nrf2/ARE pathway.

Cytokines play a vital role in immune status and inflammatory response [67]. Placental oxidative stress is often associated with increased production of pro-inflammatory cytokines, including IL-1β, IL-6, and IL-8 [68]. Excessive placental inflammation is associated with several pregnancy complications, such as IUGR and stillbirth [69]. A recent study showed that melatonin reverses the increase in IL-6 and TNF-α induced by hypoxia/reoxygenation in human primary villous trophoblasts [70]. In this study, maternal melatonin (36 mg/d) supplementation during pregnancy significantly decreased concentrations of placental pro-inflammatory cytokines, especially TNF-α, which was consistent with the results of a previous study in LPS-challenged mice [9]. TNF-α provokes various biological effects on placental and endometrial cell types, such as cell fusion, apoptosis, and hormone production [71]. A previous study has reported that TNF-α may inhibit the growth of trophoblast cells [72]. In addition, the increased TNF-α expression in the placenta was associated with impaired fetal development [73]. Thus, MT may improve placental and fetal growth through reducing placental inflammatory response.

During middle and late gestation, the placenta is a rapidly growing organ [74]. In this study, dietary MT supplementation increased placental weight per sow and tended to increase placental weight per fetus, indicating an increase in placental growth. The protein concentration, together with the ratio of RNA to DNA and protein to DNA in the placenta have been recognized as valuable biological parameters to determine placental growth and development [75]. DNA concentration was used as an index of hyperplasia, and the protein/DNA and RNA/DNA ratios were used as indices of hypertrophy and potential cellular protein synthetic activity, respectively [76]. In this study, maternal MT supplementation elevated the placental protein/DNA and RNA/DNA ratios, which is beneficial for the placental growth. In addition, the Ki67 protein is tightly linked to somatic cell proliferation. A rapid decrease of *Ki67* mRNA expression can be easily screened once the cell enters the non-proliferative state [77]. In the present study, maternal MT supplementation significantly decreased pro-apoptotic *Caspase-3* mRNA expression and had a tendency to increase *Ki67* mRNA expression, suggesting that placental cellular proliferation was enhanced. Supportively, data from ewes showed that maternal MT treatment had a tendency to increase placental cellular proliferation in cotyledonary tissue [78]. Previous study has found that pig placental weight is positively related to fetal weight [79]. Besides, placental weight is widely used as a parameter of placental functional capacity [80,81]. An important function of the placenta is to provide adequate oxygen and nutrients to the fetus to maintain fetal growth [5]. Previous studies have shown that MT treatment in pregnant ewes could improve oxygen supply to the fetus [82,83], and a potential mechanism may be associated with a decrease in oxidative stress and an increase in nitric oxide levels, leading to an increase in umbilical blood flow [82]. In this study, fetal blood gases were not measured; however, interestingly, the mRNA expression levels for a glucose transporter (*Glut3*), amino acid transporters (*SNAT2* and *SNAT3*), as well as peptide transporter 1 (*Pept1*) were significantly upregulated in the placentas of MT-supplemented sows. Upregulation of placental nutrient transporters can improve nutrient transfer to fetus, thus promoting fetal growth [84,85]. Similarly, a previous report in ewes has indicated that maternal MT supplementation improved fetal branched-chain amino acids uptake during maternal nutrient restriction, which could be applied to alleviate IUGR [14].

As a mitochondrial rich organ, the placenta requires a high level of ATP to support its growth and the active transport of nutrients. However, mitochondria are the main source of ATP and ROS formation, and also a target of ROS attack, which may lead to alterations in their structure and function [17]. Several studies have identified that mitochondrial dysfunction results from oxidative stress in the liver, intestine, and placenta [36,86]. In this study, the content of mtDNA and the antioxidant defense system in placenta were improved by dietary MT supplementation. Similarly, treatment of rotenone-induced impairment of porcine embryos with MT increased mtDNA content and decreased ROS generation [87]. It has been reported that abnormal mtDNA content can be indicative of mitochondrial dysfunction [88]. The NAD^+^ reduction is closely associated with the dysregulation of mitochondria and energy homeostasis [89]. Our data showed that placental mitochondrial function was increased by MT supplementation, as evidenced by increased placental ATP, NAD^+^ levels, and mtDNA content. In addition, mitochondrial complexes I and III are regarded as the major source of ROS generation [90]. Previous research has reported that elevated ROS generation would lead to the rapid loss of the activities of mitochondrial complexes [91]. Another study also showed a close link between enhanced ROS generation and reduced mitochondrial complex I activity in the hypoxic human placenta [92]. In this study, the mitochondrial complex I activity was increased by MT supplementation, suggesting a decrease in ROS formation in the placenta of MT-supplemented sows. Furthermore, the altered mtDNA amount is accompanied with changes in several transcriptional factors that participate in mitochondrial biogenesis [93]. Accumulating evidence indicates that SIRT1 activation reduces oxidative stress and stimulates mitochondrial biogenesis [94,95]. In our study, the protein abundance of SIRT1 was increased by dietary MT supplementation. Similarly, a previous study reported that MT could activate the SIRT1 pathway, thus promoting mitochondrial biogenesis and energy production [87]. Taken together, in this study, MT may play a protective role against oxidative stress-induced mitochondrial dysfunction and energy deficiency by improving mitochondrial biosynthesis.

## 5. Conclusions

In summary, our data indicated that dietary supplementation with MT in gestating sows could improve maternal–placental–fetal redox status and enhance placental growth and function, thereby improving pregnancy outcomes. The beneficial effects of MT might be closely related to ameliorating placental antioxidant status, inflammatory response, and mitochondrial dysfunction.

## Figures and Tables

**Table 1:** Effects of maternal MT supplementation during gestation on the reproductive performance of sows.

	CON	MT	*p*-Value
Litter size, *n*	8	8	
Piglets, total *n*			
Total born	115	118	
Born alive	106	113	
Mummified piglets	3	3	
Stillborn piglets	6	2	
Piglets born alive with weight < 900 g	12	3	
Average litter size, *n*			
Total born	14.38 ± 0.82	14.75 ± 0.63	0.72
Born alive	13.25 ± 0.73	14.12 ± 0.40	0.31
Mummified piglets	0.38 ± 0.26	0.38 ± 0.18	1.00
Stillborn piglets	0.75 ± 0.37	0.25 ± 0.16	0.23
Rate of born alive piglets with weight < 900 g, %	11.32	2.65	0.01

CON = control, MT = melatonin. Rate of live-born piglets with weight < 900 g = ‘the number of live-born piglets with weight < 900 g’/‘total number of live-born piglets’ × 100%. Data are expressed as means ± SEM, *n* = 8. Differences were considered significant at *p* < 0.05.

**Table 2:** Effects of maternal MT supplementation during gestation on placental DNA, RNA, and protein concentrations.

	CON	MT	*p*-Value
DNA, μg/g	76.00 ± 6.21	64.25 ± 4.16	0.14
RNA, μg/g	77.42 ± 14.33	96.05 ± 9.57	0.30
RNA/DNA	0.98 ± 0.15	1.59 ± 0.27	0.07
Protein, mg/g	12.42 ± 2.24	17.15 ± 0.71	0.07
Protein/DNA, mg/μg	0.15 ± 0.02	0.28 ± 0.02	<0.01

CON = control, MT = melatonin. Data are expressed as means ± SEM, *n* = 8. Differences were considered significant at *p* < 0.05.

## Data Availability

The data presented in this study are available in this manuscript or Supplementary Materials.

## Supplementary Materials

The following are available online at https://www.mdpi.com/article/10.3390/antiox10121867/s1. Table S1: Ingredients and composition of the control diet. Table S2: Primer and probe sequences used for the determination of mtDNA content. Table S3: Primer sequences of the target and reference genes.

## References

[1] Tan C., Ji Y., Zhao X., Xin Z., Li J., Huang S., Cui Z., Wen L., Liu C., Kim S.W. (2021). Effects of dietary supplementation of nucleotides from late gestation to lactation on the performance and oxidative stress status of sows and their offspring. Anim. Nutr..

[2] Herrera E., Ortega-Senovilla H. (2010). Maternal lipid metabolism during normal pregnancy and its implications to fetal development. Clin. Lipidol..

[3] Luo Z., Yao J., Xu J. (2021). Reactive oxygen and nitrogen species ratio regulate porcine embryo development during pre-implantation period: A mini-review. Anim. Nutr..

[4] Pereira A.C., Martel F. (2014). Oxidative stress in pregnancy and fertility pathologies. Cell Biol. Toxicol..

[5] Zhang S., Regnault T.R., Barker P.L., Botting K.J., McMillen I.C., McMillan C.M., Roberts C.T., Morrison J.L. (2015). Placental adaptations in growth restriction. Nutrients.

[6] Jauniaux E., Poston L., Burton G.J. (2006). Placental-related diseases of pregnancy: Involvement of oxidative stress and implications in human evolution. Hum. Reprod. Update.

[7] Fisher J.J., Bartho L.A., Perkins A.V., Holland O.J. (2020). Placental mitochondria and reactive oxygen species in the physiology and pathophysiology of pregnancy. Clin. Exp. Pharmacol. Physiol..

[8] Meng Q., Guo T., Li G., Sun S., He S., Cheng B., Shi B., Shan A. (2018). Dietary resveratrol improves antioxidant status of sows and piglets and regulates antioxidant gene expression in placenta by Keap1-Nrf2 pathway and Sirt1. J. Anim. Sci. Biotechnol..

[9] Lee J.Y., Li S., Shin N.E., Na Q., Dong J., Jia B., Jones-Beatty K., McLane M.W., Ozen M., Lei J. (2019). Melatonin for prevention of placental malperfusion and fetal compromise associated with intrauterine inflammation-induced oxidative stress in a mouse model. J. Pineal Res..

[10] Pang Y.W., Jiang X.L., Wang Y.C., Wang Y.Y., Hao H.S., Zhao S.J., Du W.H., Zhao X.M., Wang L., Zhu H.B. (2019). Melatonin protects against paraquat-induced damage during in vitro maturation of bovine oocytes. J. Pineal Res..

[11] Lanoix D., Beghdadi H., Lafond J., Vaillancourt C. (2008). Human placental trophoblasts synthesize melatonin and express its receptors. J. Pineal Res..

[12] Voiculescu S., Zygouropoulos N., Zahiu C., Zagrean A. (2014). Role of melatonin in embryo fetal development. J. Med. Life.

[13] Chen Y., Xu D., Wang J., Wang H., Wei L., Sun M., Wei W. (2006). Melatonin protects against lipopolysaccharide-induced intra-uterine fetal death and growth retardation in mice. J. Pineal Res..

[14] Lemley C., Camacho L., Meyer A., Kapphahn M., Caton J., Vonnahme K. (2013). Dietary melatonin supplementation alters uteroplacental amino acid flux during intrauterine growth restriction in ewes. Animal.

[15] Lemley C.O., Meyer A.M., Camacho L.E., Neville T.L., Newman D.J., Caton J.S., Vonnahme K.A. (2012). Melatonin supplementation alters uteroplacental hemodynamics and fetal development in an ovine model of intrauterine growth restriction. Am. J. Physiol. Regul. Integr. Comp. Physiol..

[16] Le Z., Dong S., Zhang R., Cai X., Gao A., Xiao R., Yu H. (2018). Placental mitochondrial biogenesis and function was slightly changed by gestational hypercholesterolemia in full-term pregnant women. J. Dev. Orig. Health Dis..

[17] Leduc L., Levy E., Bouity-Voubou M., Delvin E. (2010). Fetal programming of atherosclerosis: Possible role of the mitochondria. Eur. J. Obstet. Gynecol. Reprod. Biol..

[18] Richter H.G., Hansell J.A., Raut S., Giussani D.A. (2009). Melatonin improves placental efficiency and birth weight and increases the placental expression of antioxidant enzymes in undernourished pregnancy. J. Pineal Res..

[19] Hu C., Yang Y., Deng M., Yang L., Shu G., Jiang Q., Zhang S., Li X., Yin Y., Tan C. (2020). Placentae for Low Birth Weight Piglets Are Vulnerable to Oxidative Stress, Mitochondrial Dysfunction, and Impaired Angiogenesis. Oxid. Med. Cell. Longev..

[20] Yang Q., Dai S., Luo X., Zhu J., Li F., Liu J., Yao G., Sun Y. (2018). Melatonin attenuates postovulatory oocyte dysfunction by regulating SIRT1 expression. Reproduction.

[21] Barbaux S., Erwich J.J.H.M., Favaron P., Gil S., Gallot D., Golos T.G., Gonzalez-Bulnes A., Guibourdenche J., Heazell A., Jansson T. (2015). IFPA meeting 2014 workshop report: Animal models to study pregnancy pathologies; new approaches to study human placental exposure to xenobiotics; biomarkers of pregnancy pathologies; placental genetics and epigenetics; the placenta and stillbirth and fetal growth restriction. Placenta.

[22] Chavatte-Palmer P., Tarrade A. (2016). Placentation in different mammalian species. Ann. Endocrinol..

[23] Yuan T.-L., Zhu Y.-H., Shi M., Li T.-T., Li N., Wu G.-Y., Bazer F.W., Zang J.-J., Wang F.-L., Wang J.-J. (2015). Within-litter variation in birth weight: Impact of nutritional status in the sow. J. Zhejiang Univ. Sci. B.

[24] Tetro N., Moushaev S., Rubinchik-Stern M., Eyal S. (2018). The placental barrier: The gate and the fate in drug distribution. Pharm. Res..

[25] Dearlove B., Kind K., Gatford K., van Wettere W. (2017). Melatonin fed in early gestation increases fetal weight. Anim. Prod. Sci..

[26] NRC (2012). Nutrient Requirements of Swine.

[27] Wilson M.E., Biensen N.J., Youngs C.R., Ford S.P. (1998). Development of Meishan and Yorkshire littermate conceptuses in either a Meishan or Yorkshire uterine environment to day 90 of gestation and to term. Biol. Reprod..

[28] Wan J., Jiang F., Xu Q., Chen D., Yu B., Huang Z., Mao X., Yu J., He J. (2017). New insights into the role of chitosan oligosaccharide in enhancing growth performance, antioxidant capacity, immunity and intestinal development of weaned pigs. RSC Adv..

[29] Livingstone D., Martinez P.G., Michel X., Narbonne J., O’hara S., Ribera D., Winston G. (1990). Oxyradical production as a pollution-mediated mechanism of toxicity in the common mussel, *Mytilus edulis* L., and other molluscs. Funct. Ecol..

[30] Zhang X.-D., Zhu Y.-F., Cai L.-S., Wu T.-X. (2008). Effects of fasting on the meat quality and antioxidant defenses of market-size farmed large yellow croaker (*Pseudosciaena crocea*). Aquaculture.

[31] Özmen B., Özmen D., Erkin E., Güner İ., Habif S., Bayındır O. (2002). Lens superoxide dismutase and catalase activities in diabetic cataract. Clin. Biochem..

[32] Jia J., Zhang X., Hu Y.-S., Wu Y., Wang Q.-Z., Li N.-N., Guo Q.-C., Dong X.-C. (2009). Evaluation of in vivo antioxidant activities of Ganoderma lucidum polysaccharides in STZ-diabetic rats. Food Chem..

[33] Prasad A.S., DuMouchelle E., Koniuch D., Oberleas D. (1972). A simple fluorometric method for the determination of RNA and DNA in tissues. J. Lab. Clin. Med..

[34] Munro H., Fleck A. (1969). Analysis of tissues and body fluids for nitrogenous constituents. Mamm. Protein Metab..

[35] Lowry O.H., Rosebrough N.J., Farr A.L., Randall R.J. (1951). Protein measurement with the Folin phenol reagent. J. Biol. Chem..

[36] Hu L., Peng X., Qin L., Wang R., Fang Z., Lin Y., Xu S., Feng B., Wu D., Che L. (2018). Dietary nucleotides supplementation during the suckling period improves the antioxidative ability of neonates with intrauterine growth retardation when using a pig model. RSC Adv..

[37] Liu J., Zhang Y., Li Y., Yan H., Zhang H. (2019). L-tryptophan enhances intestinal integrity in diquat-challenged piglets associated with improvement of redox status and mitochondrial function. Animals.

[38] Hu L., Han F., Chen L., Peng X., Chen D., Wu D., Che L., Zhang K. (2018). High nutrient intake during the early postnatal period accelerates skeletal muscle fiber growth and maturity in intrauterine growth-restricted pigs. Genes Nutr..

[39] Jahnke G., Marr M., Myers C., Wilson R., Travlos G., Price C. (1999). Maternal and developmental toxicity evaluation of melatonin administered orally to pregnant Sprague-Dawley rats. Toxicol. Sci..

[40] Diekman M., Brandt K., Green M., Clapper J., Malayer J. (1992). Lack of a nocturnal rise of serum melatonin in prepubertal gilts. Domest. Anim. Endocrinol..

[41] Green M., Clapper J., Andres C., Diekman M. (1996). Serum concentrations of melatonin in prepubertal gilts exposed to either constant or stepwise biweekly alteration in scotophase. Domest. Anim. Endocrinol..

[42] Arend L.S., Knox R.V., Greiner L.L., Graham A.B., Connor J.F. (2019). Effects of feeding melatonin during proestrus and early gestation to gilts and parity 1 sows to minimize effects of seasonal infertility. J. Anim. Sci..

[43] McConnell S., Ellendorff F. (1987). Absence of nocturnal plasma melatonin surge under long and short artificial photoperiods in the domestic sow. J. Pineal Res..

[44] Diekman M.A., Arthington J.A., Clapper J.A., Green M.L. (1997). Failure of melatonin implants to alter onset of puberty in gilts. Anim. Reprod. Sci..

[45] De Almeida E.A., Di Mascio P., Harumi T., Spence D.W., Moscovitch A., Hardeland R., Cardinali D.P., Brown G.M., Pandi-Perumal S. (2011). Measurement of melatonin in body fluids: Standards, protocols and procedures. Childs Nerv. Syst..

[46] Paterson A., Maxwell C., Foldes A. (1992). Seasonal inhibition of puberty in domestic gilts is overcome by melatonin administered orally, but not by implant. Reproduction.

[47] Arck P., Hansen P.J., Mulac Jericevic B., Piccinni M.P., Szekeres-Bartho J. (2007). Progesterone during pregnancy: Endocrine–immune cross talk in mammalian species and the role of stress. Am. J. Reprod. Immunol..

[48] Albrecht E.D., Aberdeen G.W., Pepe G.J. (2000). The role of estrogen in the maintenance of primate pregnancy. Am. J. Obstet. Gynecol..

[49] He C., Wang J., Li Y., Zhu K., Xu Z., Song Y., Song Y., Liu G. (2015). Melatonin-related genes expressed in the mouse uterus during early gestation promote embryo implantation. J. Pineal Res..

[50] Zhang W., Wang Z., Zhang L., Zhang Z., Chen J., Chen W., Tong D. (2018). Melatonin stimulates the secretion of progesterone along with the expression of cholesterol side-chain cleavage enzyme (P450scc) and steroidogenic acute regulatory protein (StAR) in corpus luteum of pregnant sows. Theriogenology.

[51] Wang J., Zhu T., Ma X., Wang Y., Liu J., Li G., Liu Y., Ji P., Zhang Z., Zhang L. (2021). Melatonergic systems of AANAT, melatonin, and its receptor MT2 in the corpus luteum are essential for reproductive success in mammals. Biol. Reprod..

[52] Tamura H., Nakamura Y., Terron M.P., Flores L.J., Manchester L.C., Tan D.-X., Sugino N., Reiter R.J. (2008). Melatonin and pregnancy in the human. Reprod. Toxicol..

[53] Aversa S., Pellegrino S., Barberi I., Reiter R.J., Gitto E. (2012). Potential utility of melatonin as an antioxidant during pregnancy and in the perinatal period. J. Matern. Fetal Neonatal Med..

[54] Hsu C.-N., Huang L.-T., Tain Y.-L. (2019). Perinatal use of melatonin for offspring health: Focus on cardiovascular and neurological diseases. Int. J. Mol. Sci..

[55] Finkel T. (2003). Oxidant signals and oxidative stress. Curr. Opin. Cell Biol..

[56] Al-Gubory K.H., Fowler P.A., Garrel C. (2010). The roles of cellular reactive oxygen species, oxidative stress and antioxidants in pregnancy outcomes. Int. J. Biochem. Cell Biol..

[57] El-Sheikh N.M., Khalil F.A. (2011). L-Arginine and L-glutamine as immunonutrients and modulating agents for oxidative stress and toxicity induced by sodium nitrite in rats. Food Chem. Toxicol..

[58] Valko M., Rhodes C., Moncol J., Izakovic M., Mazur M. (2006). Free radicals, metals and antioxidants in oxidative stress-induced cancer. Chem. Biol. Interact..

[59] Okatani Y., Wakatsuki A., Kaneda C. (2000). Melatonin increases activities of glutathione peroxidase and superoxide dismutase in fetal rat brain. J. Pineal Res..

[60] Wakatsuki A., Okatani Y., Shinohara K., Ikenoue N., Kaneda C., Fukaya T. (2001). Melatonin protects fetal rat brain against oxidative mitochondrial damage. J. Pineal Res..

[61] Melchiorri D., Reiter R.J., Sewerynek E., Chen L.D., Nistic G. (1995). Melatonin reduces kainate-induced lipid peroxidation in homogenates of different brain regions. FASEB J..

[62] Lanoix D., Guérin P., Vaillancourt C. (2012). Placental melatonin production and melatonin receptor expression are altered in preeclampsia: New insights into the role of this hormone in pregnancy. J. Pineal Res..

[63] Vatish M., Steer P., Blanks A., Hon M., Thornton S. (2010). Diurnal variation is lost in preterm deliveries before 28 weeks of gestation. BJOG Int. J. Obstet. Gynaecol..

[64] Lanoix D., Lacasse A.A., Reiter R.J., Vaillancourt C. (2013). Melatonin: The watchdog of villous trophoblast homeostasis against hypoxia/reoxygenation-induced oxidative stress and apoptosis. Mol. Cell Endocrinol..

[65] Teixeira T.M., da Costa D.C., Resende A.C., Soulage C.O., Bezerra F.F., Daleprane J.B. (2017). Activation of Nrf2-antioxidant signaling by 1,25-dihydroxycholecalciferol prevents leptin-induced oxidative stress and inflammation in human endothelial cells. J. Nutr..

[66] Dinkova-Kostova A.T., Talalay P. (2010). NAD (P) H: Quinone acceptor oxidoreductase 1 (NQO1), a multifunctional antioxidant enzyme and exceptionally versatile cytoprotector. Arch. Biochem. Biophys..

[67] Praveena P.E., Periasamy S., Kumar A., Singh N. (2010). Cytokine profiles, apoptosis and pathology of experimental Pasteurella multocida serotype A1 infection in mice. Res. Vet. Sci..

[68] Xie C., Wu X., Long C., Wang Q., Fan Z., Li S., Yin Y. (2016). Chitosan oligosaccharide affects antioxidant defense capacity and placental amino acids transport of sows. BMC Vet. Res..

[69] Bartha J.L., Romero-Carmona R., Comino-Delgado R. (2003). Inflammatory cytokines in intrauterine growth retardation. Acta Obstet. Gynecol. Scand..

[70] Sagrillo-Fagundes L., Assunção Salustiano E.M., Ruano R., Markus R.P., Vaillancourt C. (2018). Melatonin modulates autophagy and inflammation protecting human placental trophoblast from hypoxia/reoxygenation. J. Pineal Res..

[71] Haider S., Knöfler M. (2009). Human tumour necrosis factor: Physiological and pathological roles in placenta and endometrium. Placenta.

[72] Hunt J., Atherton R., Pace J. (1990). Differential responses of rat trophoblast cells and embryonic fibroblasts to cytokines that regulate proliferation and class I MHC antigen expression. J. Immunol..

[73] Holcberg G., Huleihel M., Sapir O., Katz M., Tsadkin M., Furman B., Mazor M., Myatt L. (2001). Increased production of tumor necrosis factor-α TNF-α by IUGR human placentae. Eur. J. Obstet. Gynecol. Reprod. Biol..

[74] Krombeen S.K., Bridges W.C., Wilson M.E., Wilmoth T.A. (2019). Factors contributing to the variation in placental efficiency on days 70, 90, and 110 of gestation in gilts. J. Anim. Sci..

[75] Zhang H., Sun L., Wang Z., Deng M., Nie H., Zhang G., Ma T., Wang F. (2016). N-carbamylglutamate and L-arginine improved maternal and placental development in underfed ewes. Reproduction.

[76] Scheaffer A., Caton J., Redmer D., Arnold D., Reynolds L. (2004). Effect of dietary restriction, pregnancy, and fetal type on intestinal cellularity and vascularity in Columbia and Romanov ewes. J. Anim. Sci..

[77] Bullwinkel J., Baron-Lühr B., Lüdemann A., Wohlenberg C., Gerdes J., Scholzen T. (2006). Ki-67 protein is associated with ribosomal RNA transcription in quiescent and proliferating cells. J. Cell. Physiol..

[78] Eifert A.W., Wilson M.E., Vonnahme K.A., Camacho L.E., Borowicz P.P., Redmer D.A., Romero S., Dorsam S., Haring J., Lemley C.O. (2015). Effect of melatonin or maternal nutrient restriction on vascularity and cell proliferation in the ovine placenta. Anim. Reprod. Sci..

[79] Town S.C., Patterson J.L., Pereira C.Z., Gourley G., Foxcroft G.R. (2005). Embryonic and fetal development in a commercial dam-line genotype. Anim. Reprod. Sci..

[80] Salafia C., Charles A., Maas E. (2006). Placenta and fetal growth restriction. Clin. Obstet. Gynecol..

[81] Rumball C., Harding J., Oliver M., Bloomfield F. (2008). Effects of twin pregnancy and periconceptional undernutrition on maternal metabolism, fetal growth and glucose–insulin axis function in ovine pregnancy. J. Physiol..

[82] Sales F., Peralta O.A., Narbona E., Mccoard S.A., Gonzalezbulnes A., Parraguez V.H. (2019). Rapid Communication: Maternal melatonin implants improve fetal oxygen supply and body weight at term in sheep pregnancies. J. Anim. Sci..

[83] Tare M., Parkington H.C., Wallace E.M., Sutherland A.E., Lim R., Yawno T., Coleman H.A., Jenkin G., Miller S.L. (2014). Maternal melatonin administration mitigates coronary stiffness and endothelial dysfunction, and improves heart resilience to insult in growth restricted lambs. J. Physiol..

[84] Kwan S.T., King J.H., Yan J., Wang Z., Jiang X., Hutzler J.S., Klein H.R., Brenna J.T., Roberson M.S., Caudill M.A. (2017). Maternal choline supplementation modulates placental nutrient transport and metabolism in late gestation of mouse pregnancy. J. Nutr..

[85] Rosario F.J., Kanai Y., Powell T.L., Jansson T. (2013). Mammalian target of rapamycin signalling modulates amino acid uptake by regulating transporter cell surface abundance in primary human trophoblast cells. J. Physiol..

[86] Tian L., Huang J., Wen A., Yan P. (2020). Impaired Mitochondrial Function Results from Oxidative Stress in the Full-Term Placenta of Sows with Excessive Back-Fat. Animals.

[87] Niu Y.J., Zhou W., Nie Z.W., Shin K.T., Cui X.S. (2020). Melatonin enhances mitochondrial biogenesis and protects against rotenone-induced mitochondrial deficiency in early porcine embryos. J. Pineal Res..

[88] Ott M., Gogvadze V., Orrenius S., Zhivotovsky B. (2007). Mitochondria, oxidative stress and cell death. Apoptosis.

[89] Canto C., Menzies K.J., Auwerx J. (2015). NAD+ metabolism and the control of energy homeostasis: A balancing act between mitochondria and the nucleus. Cell Metab..

[90] Goguadze N., Zhuravliova E., Morin D., Mikeladze D., Maurice T. (2019). Sigma-1 receptor agonists induce oxidative stress in mitochondria and enhance complex I activity in physiological condition but protect against pathological oxidative stress. Neurotox. Res..

[91] Gardner P.R., Nguyen D., White C.W. (1994). Aconitase is a sensitive and critical target of oxygen poisoning in cultured mammalian cells and in rat lungs. Proc. Nat. Acad. Sci. USA.

[92] Colleoni F., Padmanabhan N., Yung H.-W., Watson E.D., Cetin I., van Patot M.C.T., Burton G.J., Murray A.J. (2013). Suppression of mitochondrial electron transport chain function in the hypoxic human placenta: A role for miRNA-210 and protein synthesis inhibition. PLoS ONE.

[93] Puigserver P., Spiegelman B.M. (2003). Peroxisome proliferator-activated receptor-γ coactivator 1α (PGC-1α): Transcriptional coactivator and metabolic regulator. Endocr. Rev..

[94] Khader A., Yang W.-L., Kuncewitch M., Jacob A., Prince J.M., Asirvatham J.R., Nicastro J., Coppa G.F., Wang P. (2014). Sirtuin 1 activation stimulates mitochondrial biogenesis and attenuates renal injury after ischemia-reperfusion. Transplantation.

[95] Zhang W., Huang Q., Zeng Z., Wu J., Zhang Y., Chen Z. (2017). Sirt1 inhibits oxidative stress in vascular endothelial cells. Oxid. Med. Cell. Longev..

