# Supplementary material for: Effects of Melatonin Supplementation during Pregnancy on Reproductive Performance, Maternal–Placental–Fetal Redox Status, and Placental Mitochondrial Function in a Sow Model
Source: Antioxidants (Basel). 2021 Nov 24;10(12):1867. doi: 10.3390/antiox10121867 (PMC8698367; doi:10.3390/antiox10121867)
Supplement: Supplementary file 1 [file antioxidants-10-01867-s001.zip › antioxidants-1393207-supplementary.pdf]

Table S1. Ingredients and nutrient composition of the control diet (as fed basis)

	Control diet
Ingredients, %	
Corn	62.39
Dehulled soybean meal	13.10
Fish meal	2.00
Flour	10.00
Corn starch	10.00
L-Lys HCl (78%)	0.10
L-Threonine (98%)	0.02
Limestone	0.84
Dicalcium phosphate	0.46
Salt	0.40
Choline chloride (50%)	0.14
Mineral premix[1]	0.50
Vitamin premix[2]	0.05
Total	100.00
Nutrient composition, %[3]	
Digestible energy, Mcal/kg	3.36
Crude protein	13.39
Calcium	0.60
Available phosphorus	0.27
Lysine	0.60
Methionine	0.21
Threonine	0.46
Tryptophan	0.14

[1] Mineral premix provided per kilogram of diet: Cu 10 mg, Fe 100 mg, I 0.6 mg, Zn 100 mg, Mn 30 mg, Se 0.25 mg.

[2] Vitamin premixe provided per kilogram of diet: VA 7500IU, VD_3 5000IU, VE 37.5IU, VK_3 5 mg, VB_1 5 mg, VB_2 12.5 mg, VB_6 7.5 mg, VB_{12} 0.05 mg, biotin 0.2 mg, niacin 50 mg, folic acid 2.5 mg, D-calcium pantothenate 25 mg, ethoxyquinoline 0.25 mg.

[3] Nutrient levels were calculated.

Table S2. Primer and probe sequences used for determination of mtDNA content.

Gene	Genebank accession	Primer sequence	Product size (bp)
Mitochondrial D-loop	AF276923	5’-GATCGTACATAGCACATATCATGTC-3’	198
		5’-GGTCCTGAAGTAAGAACCAGATG-3’	
		5’- (FAM) CCAGTCAACATGCGTATCACCACCA(Eclipse) -3’	
β-actin	DQ452569	5’-CCCCTCCTCTCTTGCCTCTC -3’	74
		5’-AAAAGTCCTAGGAAAATGGCAGAAG -3’	
		5’- (FAM) TGCCACGCCCTTTCTCACTTGTTCT (Eclipse) -3’	

Table S3. Primer sequences of the target and reference genes.

Gene	Genebank accession	5'-Primer (F)	3' -Primer (R)	Product size (bp)
SOD	NM_001190422.1	TCCATGTCCATCAGTTTGGA	AGTCACATTGCCCAGGTCTC	131
CAT	NM_214301.2	ACGCCTGTGTGAGAACATTG	GTCCAGAAGAGCCTGAATGC	124
GPX1	NM_214201.1	AAATGCTCACCCGCTCTTC	GTCATTGCGACACACTGGAG	118
Nrf2	XM_003133500.5	GCCCCTGGAAGCGTTAAAC	GGACTGTATCCCCAGAAGGTTGT	67
HO1	NM_001004027.1	AGGCTGAGAATGCCGAGTTC	TGTGGTACAAGGACGCCATC	90
NQO1	NM_001159613.1	CCAGCAGCCCGGCCAATCTG	AGGTCCGACACGGCGACCTC	160
Bax	XM_003127290.4	CTGACGGCAACTTCAACTGG	CGTCCCAAAGTAGGAGAGGA	200
Bcl2	XM_003121700.2	AGCATGCGGCCTCTATTTGA	GGCCCGTGGACTTCACTTAT	120
Caspase3	NM_214131.1	GTGGGACTGAAGATGACA	ACCCGAGTAAGAATGTG	190
Ki67	XM013983877.1	GTCCTCAACAAGCCCGAGAA	ATCTTCCTTCCCGGATGGCT	309
Glut1	XM_021096908.1	GCCTGAGACCAGTTGAAAGCAC	CTGCTTAGGTAAAGTTACAGGAG	155

Glut3	XM_021092391.1	TGCACGGGCTTTGTGCCGATG	AAGGAGGTGAAGATTAGGAA	132
SNAT1	XM_003355629	AAGAACCTGGGCTATCTCGG	TGTTGCGTTAGGACTCGTTG	138
SNAT2	NM_018976	GTTACCTTTGGTGATCCAGGC	ACCAATGACACCAGCAGAACC	96
SNAT3	XM_003355630	GTTCTTTGCCTTCACTCACTA	GACCCAAGCCTCCAGATT	186
Pept1	NM_214347	CCCAGGCTTGCTACCCAC	ACCCGATGCACTTGACGA	144
MT1	XM_021078041.1	GGTGGCTGTGTATCCGTACC	TGACACTCAAGCCCATCAGG	110
β-actin	DQ845171.1	GGCGCCCAGCACGAT	CCGATCCACACGGAGTACTTG	66

Glut1=Slc2a1, Glut3=Slc2a3, SNAT1=Slc38a1, SNAT2=Slc38a2, SNAT3=Slc38a4.
